# Association between water and sanitation and soil-transmitted helminthiases: Analysis of the Brazilian National Survey of Prevalence (2011–2015)

**DOI:** 10.1186/s13690-021-00602-7

**Published:** 2021-05-19

**Authors:** Kasandra Isabella Helouise Mingoti Poague, Sueli Aparecida Mingoti, Léo Heller

**Affiliations:** 1 Department of Sanitary and Environmental Engineering, Federal University of Minas Gerais, Av. Pres. Antônio Carlos, 6627 - Pampulha, Minas Gerais 31270-901 Belo Horizonte, Brazil; 2 Department of Statistics, Federal University of Minas Gerais, Belo Horizonte, Brazil; 3 René Rachou Institute, The Oswaldo Cruz Foundation in the State of Minas Gerais, Belo Horizonte, Minas Gerais Brazil

**Keywords:** Ascaris lumbricoides, Trichuris trichiura, Hookworm, Water, sanitation and hygiene (WASH), Risk factors, Schoolchildren, Brazil

## Abstract

**Background:**

Most of the studies conducted in Brazil assessing the relationship between water, sanitation and hygiene (WASH) and Soil-transmitted helminth (STH) infections, have focused on cases, reflecting the reality of small areas and not of a countrywide situation. In order to fill this gap, the current paper presents an epidemiological study exploring the association between water and sanitation and STHs prevalence in students from 7 to 17 years old, in all 27 Brazilian Federation Units.

**Methods:**

Three ecological studies were carried out considering the prevalence of ascariasis, trichuriasis, and hookworm as outcome variables. The sample consisted of 197,567 students aged 7–17 years old living in 521 Brazilian municipalities. Data were retrieved from the National Survey on the Prevalence of Schistosomiasis mansoni and Soil-transmitted helminth infections (2011–2015). The Generalized Linear Model with the negative binomial distribution was used to evaluate the statistical association between outcomes and explanatory variables. Univariate and Multivariate analyses were conducted with 25 and 5 % significance levels, respectively. Data were aggregated considering municipalities as the geographical unit for analysis.

**Results:**

Protective association was found between access to filtered water and adequate sanitation in schools with ascariasis (RR 0.989, CI 95 % 0.983–0.996; RR 0.988, CI 95 % 0.977–0.998), access to filtered water in schools with trichuriasis (RR 0.986, CI 95 % 0.979–0.993) and adequate sanitation at home with hookworm ((RR 0.989, CI 95 % 0.982–0.996). The percentage of population served with *Bolsa Família* Program, used as a proxy for poverty, was the only significant variable common to all models.

**Conclusions:**

Our findings support that WASH, both in schools and homes, are essential to schoolchildren health with regard to STHs. However, sanitary interventions will not be fully effective in preventing STH infections without promoting access to quality public services, particularly for people living in poverty, the most vulnerable group.

**Supplementary Information:**

The online version contains supplementary material available at 10.1186/s13690-021-00602-7.

## Background

Present in the daily life of humans since prehistoric times, intestinal parasites are among the most common infections worldwide. According to data published by the World Health Organization (WHO), in 2016, about a quarter of the world’s population (over 1.5 billion people) was infected with soil-transmitted helminth (STH) infections - ascariasis, hookworm and trichuriasis [1].

The prevalence and distribution of STHs are the result of the interdependence of human factors (social, economic and cultural), environmental conditions (temperature, humidity, soil, etc.) and biological aspects of the helminths [2]. In spite of their low mortality rates, these diseases constitute a serious public health problem due to the long-term effects on malnutrition, on compromising children’s physical and intellectual development, and on the reduction of adult work productivity [2].

Particularly in Brazil, according to WHO, in 2018, 9,475,765 Brazilian children (aged 0–15 years) required preventive chemotherapy for STHs [3]. However, this estimate was based on surveys with partial coverage. Also, in Brazil there is a lack of statistical data showing the real national prevalence of these parasites. Most of the information are provided from cases reflecting only the reality of small areas, therefore, not representing the whole country’s situation [4, 5]. Only nationwide surveys can contribute to a more accurate picture of diseases spread and their distribution within Brazilian territory [6].

With the objective of increasing knowledge and providing support for planning more precise public policies for control of these infections, the first National Survey on the Prevalence of Schistosomiasis mansoni and Soil-transmitted helminth infections (2011–2015) in Brazil was carried out covering all the 27 Brazilian Federation Units [6]. It was a population-based cross-sectional study with the objective of determining the current prevalence of schistosomiasis mansoni, trichuriasis, hookworm and ascariasis, in students from 7 to 17 years old. For this purpose, 197,564 students living in 521 municipalities distributed in all five geographical regions of the country were examined [6].

Results of the National Survey published in early 2018 indicated a reduction in STHs prevalences compared to those observed in formers surveys with partial coverage conducted in the country (6, see also Supplementary Material). Nevertheless, high rates of prevalence (greater than 20 %) were found in municipalities of the North and Northeast regions of the country [6].

The reduction of STHs prevalences along the past decades in Brazil and the current disparities in prevalence among municipalities raise questions on the determinants of the control of these diseases. Since they are conditioned by a multiplicity of factors, a key hypothesis for this new epidemiological profile would be the role of environmental determinants, particularly the access to water and sanitation services. Although several studies already showed that water, sanitation and hygiene (WASH) play important role in the interruption of the transmission cycle of these parasites [4, 7, 8], empirical evidence that links WASH improvements to reduction in STH infections is still scarce [8, 9]. In this context, the present study aimed to evaluate the existence of association between water and sanitation and the prevalence of ascariasis, trichuriasis and hookworm in students from 7 to 17 years old, in the 27 Brazilian Federation Units.

## Methods

### Study design

The current epidemiological study can be classified as ecological, explanatory, observational and non-directional (cross-sectional).

### Database

Three statistical models were adjusted considering the prevalence of each STH (ascariasis, trichuriasis, and hookworm) as outcome variable. Data were retrieved by the National Survey on the Prevalence of Schistosomiasis mansoni and Soil-transmitted helminth infections (2011–2015) [6]. Municipalities were used as the geographical unity of analysis.

The National Survey was a population-based cross-sectional study conducted by the Oswaldo Cruz Foundation (FIOCRUZ) in 521 municipalities distributed in all five regions of the country, with the objective of assessing the current prevalence of schistosomiasis mansoni and STHs in schoolchildren from 7 to 17 years old. Although it was the third national STH survey carried out in the country, it was the first to cover all Brazilian states. Details about the survey, including its sample strategy can be found in Portuguese language in Katz [6] or in English in the Supplementary Material of this paper.

Due to the large size of Brazil (the largest country in South America and the fifth largest nation in the world), and thus, great distance between cities, the survey data could not be collected in all municipalities at the same time. The data collection took 4 years starting in 2011 and finishing in 2015. The survey, therefore, does not provide annually data on the prevalence of intestinal parasitic diseases. It provides instead, one prevalence value for each municipality, that represents the prevalence for the entire period (2011–2015).

The explanatory factors comprised 15 variables of water, sanitation, education, socioeconomic status and health, collected from different sources of public database. These variables were chosen by crossing the determinants of STH infections indicated in the literature with the public data available by the Brazilian government. Relevant factors related to the diseases such as deworming cover, collective wastewater treatment, hygiene practices could not be included, because there were no sufficient data available for all municipalities in the study. The explanatory variables were grouped as follows:

(i) Variables from the National Survey related to the sampling strategy. The endemic level for schistosomiasis mansoni (non endemic, low and high endemicity) was inserted as a nominal categorical variable (0, 1 and 2, respectively) and population size introduced as the natural logarithm of the population (number of inhabitants) of each municipality in 2010. The first variable was provided by FIOCRUZ while the second by the Brazilian National Census. Those two variables were kept in the statistical analyses in order to minimize possible sampling bias (more information can be found in the Supplementary Material); (ii) Main explanatory variables (see Table 1) expressing the water and sanitary conditions in households as well as in schools. These variables were designed according to the definitions adopted by the National Plan of Basic Sanitation (PLANSAB) for adequate water and sanitation services [10]; (iii) Explanatory variables (see Table 2) included to control confounding effects, so that the associations between the response and the main explanatory variables would not be spurious.

Since data on the prevalence of STHs (outcome variables) refer to a five-year interval, the year 2013 (the midpoint of 2011–2015 period) was adopted as a reference period for the collection of explanatory variables data. Considering that only in 2014 the National School Census started to cover schools from all Brazilian states, data from 2014 were used as a reference for all explanatory variables related to school (see Table 1).

**Table 1:** Description of the main explanatory variables

Variable (%)	Description	Period	Source
Population served with adequate water supply at home	Population supplied with piped water in at least one of the rooms of their household from the following sources: general distribution network, well or spring water on or off the property	2013*	IBGE
Population served with adequate sanitation at home	Population that disposes its sewage in a general sewerage system, rainwater drainage or septic tank	2013*	IBGE
Population served with household solid waste collection	Population served with direct and indirect household solid waste collection, regardless of location (urban or rural)	2013*	IBGE
Schools with filtered water	Schools that provide their students with filtered water from filters, such as clay, crockery, plastic or activated carbon filters, that typically has porous filter elements to retain impurities	2014	INEP
Schools served with adequate water supply	Schools with adequate water supply, i.e. which are provided with public network, artesian well and “cacimba” / well / cistern	2014	INEP
Schools served with adequate sanitation	Schools that dispose their sewage into a public sewerage system or septic tank	2014	INEP
Schools with proper solid waste disposal	Schools whose solid waste produced is periodically collected or recycled	2014	INEP

*Values ​​were obtained through population projections. *IBGE * Brazilian Institute of Geography and Statistics, *INEP *National Institute for Educational Studies and Research Anísio Teixeira.

**Table 2:** Description of the explanatory confounder variables

Variable	Description	Period	Source
% Municipality’s urbanization	Percentage of municipality’s inhabitants living in urban areas	2013*	IBGE
% Population served with *Bolsa Família* Program - BFP	Percentage of municipality’s population served by BFP (used as a poverty and social inequality identifier)	2013	MDS
% Population served by primary health care services	Percentage of municipality’s population attended by Family Health Teams and Primary Care Teams	2013	MS
% Population employed in agricultural sector − 18 years and over	Percentage of active population (18 years old and over) of each municipality employed in the agricultural sector	2013*	IBGE
Municipal Human Development Index: Education Component	Proxy of municipality’s population access to education. Its value ranges from 0 to 1	2013*	IBGE
Region	Country region where the municipality is inserted: 0 - Northeast, 1 - North, 2 - Southeast, 3 - South, 4 - Midwest, 5 - Federal District	-	IBGE

*Variables whose values ​​were obtained through population projections, *MS* Ministry of Health, *MDS* Ministry of Social Development.

For all other explanatory variables, with exception of “% Population served by primary health care services” and “% Population served by *Bolsa Família* Program - BFP”, projections were performed using linear and geometric growth models, which are appropriate to short-term estimates [11]. Data from the 1991, 2000 and 2010 Brazilian National Census, conducted by IBGE, were used to estimate the values for the year 2013 (see Tables 1 and 2).

The variable “% Population served by *Bolsa Família* Program - BFP” (see Table 2) was used in this study as a proxy for poverty. The BFP is a conditional direct cash transfer government program, created in 2003. It serves families living in poverty and extreme poverty situation, namely: families with per capita income in Brazilian currency up to R$ 89.00 per month (about US$ 22); families with pregnant women, children or adolescents from 0 to 17 years old, with per capita income between R$ 89.00 and R$ 178.00 per month (US$ 22 to US$ 44).

### Statistical Analysis

The Generalized Linear Model (GLM) with the negative binomial distribution was used to evaluate the statistical association between outcomes and explanatory variables. The negative binomial distribution was adopted in order to minimize overdispersion in the regression models, a phenomenon that when not controlled, may result in spurious associations, i.e. an explanatory variable might be seen as a significant predictor in the analysis when it is not [12].

Variables with significance level (p-value) less than 0.25 in the univariate analysis were kept to compose the multivariate model as suggested by Bendel and Afifi [13]. Multivariate analyses were performed in sequential steps following the stepwise statistical method to allow the progressive elimination of explanatory variables that were not statistically significant at level of 5 % [12].

Interaction variables were assessed by constructing a new variable composed by multiplying the two independent variables that were suspected of interaction [12]. The significance level of 5 % was used as a criterion for the interaction inclusion in the final model. All analyses were performed using the Stata software, version 14 (Stata Corporation, College Station, TX, USA).

The goodness of fit analysis of the models was performed by using the following statistics: Pearson’s deviance, natural logarithm of the likelihood function (log-likelihood), Akaike (AIC) and Bayesian (BIC) information criteria. Possible outliers were identified by graphical analysis of Pearson residuals from the models and the Cook’s distance criterion [14]. As suggested in Stata manuals, any point with Cook’s distance greater than 4/n, where n is the number of observations, was examined [15].

In order to reduce the overdispersion phenomenon, robust variance estimation combined with quasi-likelihood variance multipliers were employed for ascariasis and trichuriasis multivariate models, as proposed by Hilbe [12]. For hookworm only robust variance estimation was used.

Regardless of the statistical association (p-value) found in univariate analyses, the region factor was kept in the multivariate analysis since, although indirectly, it introduces information on specific cultural feature of each region of the country. Each Brazilian geographic region presents distinct physical characteristics resulting from its location, such as climatic conditions and soil type, which could interfere with the disease transmission cycle. Also, the regions differ administratively in the way governments promote public policies. It is important to point out that absence of variables expressing cultural factors were raised, throughout the literature review, as a limitation of studies on risk factors of the occurrence of worm diseases [16].

## Results

### Database Description

The minimum prevalence for all diseases was 0 %, reaching a maximum of 77.38 %, 91.37 and 54.12 % for ascariasis, trichuriasis, and hookworm, respectively. The average prevalence, median prevalence and standard deviation for each intestinal parasitosis were: 5.76 %, 1.94 %, 9.39 % for ascariasis; 5.34 %, 1.43 %, 10.08 % for trichuriasis; 2.74 %, 0.39 and 6.35 % for hookworm.

Most municipalities were small or medium-size, with 41.65 % comprising population between 20,000 and 150,000 inhabitants, followed by 39.92 % with population under 20,0000 inhabitants. Only 12.09 % had population between 150,000 and 500,000 inhabitants and 6.33 % over 500,000 inhabitants. The majority of the municipalities that participated in the National Survey were located in the Northeast region (43.38 %) of the country, followed by Southeast (22.27 %), North (15.74 %), South (10.17 %), Midwest (8.25 %) and Federal District (0.19 %). Regarding endemic level for schistosomiasis mansoni, only a small percentual of the municipalities were in endemic areas (16.32 % in low and 12.28 % in high endemic).

The descriptive statistics of both main and confounding explanatory variables are presented in Table 3. In general, the results exposed the heterogeneity of the municipalities population in terms of access to public services and infrastructure.

**Table 3:** Descriptive statistics for continuous explanatory variables (n = 521)

Variable	Mean	Standard deviation	Minimum	Maximum
% Population served with adequate water supply at home	82.97	17.98	11.51	100.00
% Population served with adequate sanitation at home	42.61	31.51	0.02	99.96
% Population served with household solid waste collection	73.53	23.03	0.00	99.91
% Schools with filtered water	87.80	21.91	0.00	100.00
% Schools served with adequate water supply	89.17	19.76	95.24	100.00
% Schools served with adequate sanitation	95.26	13.19	16.67	100.00
% Schools with proper solid waste disposal	67.80	31.54	1.27	100.00
% Municipality’s urbanization	68.41	23.20	13.67	100.00
% Population served with *Bolsa Familia* Program – BFP	38.99	21.99	1.22	100.00
% Population served by primary health care services	82.51	23.90	0.06	100.00
% Population employed in agricultural sector − 18 years and over	28.52	20.29	0.18	92.99
Municipal Human Development Index: Education Component	0.61	0.10	0.31	0.85

### Univariate Analysis

In the univariate statistical analysis (see Table 4) all main and confounding explanatory variables were statistically significant at a significance level of 25 %, except “% Population served by primary health care services”, for ascariasis prevalence (*p*-value 0.934).

Among the main explanatory and confounding variables, “% Population served by *Bolsa Família* Program - BFP” was the most strongly associated with the prevalence of each disease (RR 1.028, CI 75 % 1.028–1.032, *p*-value < 0.001 for ascariasis; RR 1.026, CI 75 % 1.022–1.030, *p*-value < 0.001 for trichuriasis; RR 1.053, CI 75 % 1.048–1.057, *p*-value < 0.001 for hookworm).

**Table 4:** Univariate analysis results for each STH outcome (*n* = 521)

	Ascariasis		Trichuriasis		Hookworm		
Variable	*P*-value	RR (CI 75 %)	*P*-value	RR (CI 75 %)	*P*-value		RR (CI 75 %)
Non endemic (0)	Reference						
Low (1)	0.082	0.736 (0.601–0.902)	0.011	0.598 (0.474–0.755)	0.987*		1.004 (0.773–1.303)
High (2)	0.052	1.469 ( 1.170–1.844)	0.012	1.767 (1.362–2.293)	0.029		1.744 (1.301–2.336)
Natural log of Population in 2010	0.558*	0.972 (0.919–1.028)	0.800*	0.986 (0.925–1.051)	< 0.001		0.778 (0.723–0.837)
Northeast Region (0)	Reference						
North Region (1)	0.564*	1.104 (0.906–1.347)	0.026	1.558 (1.238–1.961)	0.302*	0.801 (0.625–1.026)	
Southeast Region (2)	< 0.001	0.285 (0.238–0.341)	< 0.001	0.316 (0.256–0.390)	< 0.001	0.177 (0.142–0.221)	
South Region (3)	< 0.001	0.317 (0.247–0.405)	0.001	0.431 (0.325–0.571)	< 0.001	0.020 (0.012–0.033)	
Midwest Region (4)	< 0.001	0.095 (0.067–0.136)	< 0.001	0.048 (0.030–0.077)	< 0.001	0.082 (0.053–0.126)	
Federal District Region (5)	0.037	0.063 (0.014–0.289)	0.013	0.018 (0.003–0.113)	0.019	0.018 (0.002–0.127)	
% Population served with adequate water supply at home	< 0.001	0.974 (0.967–0.981)	< 0.001	0.979 (0.975–0.983)	< 0.001	0.957 (0.952–0.962)	
% Population served with adequate sanitation at home	< 0.001	0.988 (0.986–0.991)	< 0.001	0.991 (0.988–0.994)	< 0.001	0.974 (0.971–0.977)	
% Population served with household solid waste collection	< 0.001	0.987 (0.984–0.990)	0.008	0.991 (0.988–0.995)	< 0.001	0.971 (0.967–0.975)	
% Schools with filtered water	< 0.001	0.987 (0.983–0.990)	< 0.001	0.983 (0.980–0.987)	0.08	0.993 (0.988–0.997)	
% Schools served with adequate water supply	< 0.001	0.985 (0.981–0.988)	< 0.001	0.984 (0.980–0.988)	0.168	0.994 (0.989–0.999)	
% Schools with proper solid waste disposal	< 0.001	0.988 (0.986–0.991)	< 0.001	0.991 (0.988–0.993)	< 0.001	0.977 (0.974–0.980)	
% Schools served with adequate sanitation	< 0.001	0.977 (0.971–0.983)	< 0.001	0.977 (0.971–0.983)	0.01	0.976 (0.968–0.985)	
% Municipality’s urbanization	0.002	0.991 (0.987–0.994)	0.037	0.993 (0.989–0.997)	< 0.001	0.976 (0.969–0.983)	
% Population served by *Bolsa Família* Program	< 0.001	1.028 (1.025–1.032)	< 0.001	1.026 (1.022–1.030)	< 0.001	1.053 (1.048–1.057)	
% Population served by primary health care services	0.934*	1 (0.997–1.004)	0.218	0.966 (0.992–1.000)	< 0.001	1.019 (1.015–1.023)	
% Population employed in the agricultural sector − 18 years and over	< 0.001	1.014 (1.010–1.018)	0.01	1.010 (1.005–1.014)	< 0.001	1.030 (1.025–1.035)	
Municipal Human Development Index: Education Component	< 0.001	0.015 (0.008–0.030)	< 0.001	0.019 (0.008–0.040)	< 0.001	0.001 (0.0005–0.003)	

*Not statistically significant at 25 % level. *RR* Rate Ratio

### Multivariate Analysis

Table 5 presents the results from multivariate analysis. The significant explanatory variables (at 5 % level) for ascariasis were: “% Schools with filtered water”, “% Schools served with adequate sanitation”; “% Population served by *Bolsa Família* Program” and “% Municipality’s urbanization”. The results were similar for trichuriasis but the final model also included the variable “% Population served by primary health care services” and did not include the variable “% Schools served with adequate sanitation”.

**Table 5:** Results from multivariate analysis for the outcomes ascariasis, trichuriasis and hookworm prevalence (*n* = 521)

	Ascariasis		Trichuriasis		Hookworm	
Variable	RR (CI 95 %)	*P*-value	RR (CI 95 %)	*P*-value	RR (CI 95 %)	*P*-value
Non endemic– 0	Reference					
Low – 1	0.765 (0.562–1.042)	0.089*	0.693 (0.481–0.998)	0.048	0.994 (0.681–1.451)	0.973*
High – 2	1.226 (0.859–1.751)	0.262*	1.667 (1.096–2.536)	0.017	1.570 (1.016–2.426)	0.042
Natural log of Population in 2010	1.125 (1.016–1.246)	0.024	1.037 (0.912–1.181)	0.577*	1.180 (1.035–1.345)	0.013
Northeast Region – 0	Reference					
North Region – 1	0.630 (0.414–0.959)	0.031	0.915 (0.589–1.424)	0.695*	0.999 (0.660–1.512)	0.996*
Southeast Region – 2	0.523 (0.347–0.787)	0.002	0.463 (0.282–0.760)	0.002	0.448 (0.276–0.726)	0.001
South Region – 3	0.520 (0.288–0.939)	0.030	0.551 (0.278–1.094)	0.089*	0.107 (0.042–0.270)	< 0.001
Midwest Region − 4	0.186 (0.095–0.364)	< 0.001	0.068 (0.028–0.164)	< 0.001	0.262 (0.117–0.587)	0.001
Federal District Region – 5	0.067 (0.006–0.811)	0.034	0.012 (0.001–0.256)	0.005	0.044 (0.002–1.076)	0.055*
% Schools with filtered water	0.989 (0.983–0.996)	0.001	0.986 (0.979–0.993)	< 0.001	-	-
% Schools served with adequate sanitation	0.988 (0.977–0.998)	0.019	-	-	-	-
% Municipality’s urbanization	1.008 (1.000–1.015)	0.045	1.014 (1.005–1.022)	0.002	-	-
% Population served by *Bolsa Família* Program	1.021 (1.010–1.032)	< 0.001	1.022 (1.009–1.034)	0.001	1.031 (1.018–1.043)	< 0.001
% Population served by primary health care services	-	-	0.990 (0.983–0.997)	0.006	-	-
% Population served with adequate sanitation at home	-	-	-	-	0.989 (0.982–0.996)	0.003
Model’s constant (intercept)	0.0422 (0.0062–0.2875)	0.001	0.050 (0.0064–0.394)	0.004	0.0018 ( 0.0003–0.0102)	< 0.001

* Not statistically significant at 5 % level. *RR* Rate Ratio

In the case of hookworm, the multivariate model included: “% Population served with adequate sanitation at home” and “% Population served by *Bolsa Família* Program”.

No interaction was statistically significant with the outcome variables and therefore, they were discarded. The “% Population served by *Bolsa Família* Program” was the only significant explanatory variable common to the three models.

### Goodness of fit

Cook’s distance and Pearson’s residual analysis of the three models pointed to the presence of 21, 27 and 24 possible outliers for ascariasis, trichuriasis and hookworm, respectively. After thorough investigation it became clear that these points corresponded to the highest prevalence rates reported by the National Survey. Observing the prevalence sample distribution, the amount of municipalities listed as possible outliers with values above the respective 75 % percentile (PC) were: 19 (PC = 7.80 %) for ascariasis, 23 (PC = 6.25 %) for trichuriasis, and 19 (PC = 2.38 %) for hookworm. Regards to location, these municipalities were homogeneously distributed among all five Brazilian regions.

Multivariate models were adjusted excluding the municipalities detected as possible outliers and keeping the same explanatory variables previously included (see Table 5). The composition of ascariasis and hookworm models remained unchanged (*p*-values less than 5 %). However, when dealing with trichuriasis as the outcome, the p-value of the variable “% Population served by primary health care services” increased to 0.079, not being significant at 5 %. After these analyses, it was decided to keep the models adjusted with all data since removing the highest prevalence values would mischaracterize the outcome variables profile.

## Discussion

The results from the statistical models indicated that water in schools is related to the occurrence of parasitic infections caused by *A.lumbricoides* and *T.Trichiura* as a protective factor. For 1 % increase in availability of filtered water in schools the models showed an effect of 1.1 and 1.4 % decrease in the mean of ascariasis and trichuriasis prevalence, respectively (RR 0.989, CI 95 % 0.983–0.996; RR 0.986, CI 95 % 0.979–0.993). With regards to ascariasis, the increase of 1 % in schools served with adequate sanitation also provides a decrease of 1.2 % in the mean of the infection prevalence (RR 0.988, CI 95 % 0.977–0.998).

Besides preventing transmission through ingestion of contaminated water with infective eggs, access to safe water also enables the adoption of hygiene practices which play an important role in breaking the transmission cycle, even with deficiencies in sanitation infrastructure in homes and schools [7, 17, 18].

According to Brazilian legislation on education [19], elementary and high school students must meet a minimum of 4 school-hours per day. For high school this dedication should be progressively increased to seven hours daily [19]. Although this amount of time represents between 17 and 29 % of a day, it is during this period that children receive school meals, a possible disease transmission route, since food safety depends on the sanitary conditions of the place where the meals are prepared and served. School meals are the main and sometimes the only meal of the day guaranteed for most Brazilian children, especially in contexts of vulnerability [20]. Due to this fact, there are students who eat any meal offered at school, regardless of the menu and the quality of the food offered [20].

*A. lumbricoides* eggs have a great ability to adhere to surfaces. Once present in the environment or in food, these eggs are not easily removed by washing [2]. Previous studies indicated that inadequate hygiene procedures of food handlers were also associated with precarious working conditions and non-exclusive personnel in the cooking area, since individuals who handled food in schools also performed others activities, such as cleaning toilet [21].

Regarding hookworm, the multivariate model showed a different trend for WASH variables, remaining only one variable related to household sanitation conditions (RR 0.989, CI 95 % 0.982–0.996). According to the model (see Table 5), 1 % increase in the population served with adequate sanitation at home would result in 1.1 % decrease in the mean of hookworm prevalence.

Hookworm transmission occurs mainly through penetration of the skin by infective larvae, generally when children are walking barefoot [2]. In general, even if a child attends a school that has poor sanitation infrastructure, the exposure route through skin contact is minimized because the social praxis in the Brazilian culture is that usually, children wear shoes at school. At home, it depends on the dynamics of each family the permission to walk barefoot.

Even though our findings support that WASH (both in schools and homes) are important protective factors against STHs, the variable “% Population served by *Bolsa Família* Program - BFP”, used as a proxy of poverty, was the most strongly associated with the prevalence of each disease and the only variable presented in all three models. Other studies had also shown, with different proxies, that poverty plays a key role in the prevalences of STHs [4, 22, 23].

The results found in the current study reinforce that sanitation interventions will not be fully effective in controlling parasite infections without public policies that promote the population access to quality public services and also that take into account the structural social inequalities. As previously reported in some cluster-randomized trials [24–26], improvements in household and school sanitation alone were insufficient to reduce and control STH infections.

The results are also of concern when considering the Brazilian scenario: according to the eligibility criteria adopted by the World Bank [27], a quarter of the country’s population (26.5 % of the inhabitants, or almost 55 million people) live below the poverty line [27]. Moreover, among the Brazilian population aged 0–14 years, precisely the group most susceptible to parasitic infections, approximately 43.4 % **(**23.8 million), live in poverty [27].

The variable “% Municipality’s urbanization” in the ascariasis and trichuriasis prevalence models presented RR values greater than 1 (RR 1.008, 95 % CI: 1.000–1.015; RR 1.014, 95 % CI 1.005–1.022, respectively). In other words, the higher the percentage of the urban population in the municipality, the higher the expected mean prevalence for these diseases, as pointed out by other authors [4, 22, 28]. Although in the past, STHs were considered rural parasites, ascariasis and trichuriasis are now seen as an urban public health problem. This change is probably due to the intense rural exodus that culminated in the dense occupation of poor neighborhoods and slums in cities, where unhealthy conditions are most severe [2].

Although the transmission route of ascariasis and trichuriasis is the same (fecal-oral), the health care variable showed statistical significance only with the prevalence of trichuriasis, being identified as a protective factor (RR 0.990, CI 95 % 0.983–0.997). Despite this good result, it is important to point out that the registration sheet used by the Brazilian health care agents to take notes about the environmental characteristics needs to be improved since it is restricted to mere categorical classification, limiting the complete evaluation of the household and the sanitation services [29]. The collected data also do not allow a complete understanding of whether the normative content of the Human Right to Water and Sanitary Sewage (HRtWS) is being fulfilled at homes (quality, quantity, physical and financial accessibility, acceptability, dignity and privacy) [30].

Other important aspects related to parasites cycle cannot be computed in the form sheet, such as whether there is proper pavement or the ground of the household is exposed soil, adoption of individual hygiene, and barefoot habits. In addition, there is no mention or questioning about rain water management, the fourth basic sanitation component according to the National Basic Sanitation Law [31]. The rain water control, combined with other sanitation components, allows the reduction of transposition of ascariasis and trichuriasis eggs and limits the soil moisture content, preventing the development of hookworm larvae [2].

### Limitations

Finally, some limitations of the current study have to be highlighted. First, the nature of the present research as an ecological study, subject to the effect of ecological fallacy. Second, outcome and explanatory variables were collected in different periods of time, which could lead to underestimation of the true effects, known as regression dilution bias. Third, the influence of weather seasonality in the collection of the outcome data was not take into account. Fourth, the lack of information in the database of variables expressing sociocultural factors, hygiene practices and the qualitative dimension of the provided public services. Variables of this type should be included in future works.

## Conclusions

Although many efforts have been made to control STH infections and a lot of progress has been accomplished, these diseases continue to be part of the public health agenda in Brazil. In this context, the present study contributes substantially to the knowledge of the country’s scenario regarding parasitic diseases, since the analysed data refer to a sample of students from 7 to 17 years old, collected in municipalities from all the 27 Brazilian Federation Units.

It is also important to emphasize that in the literature, there are few epidemiological studies considering the dynamics of the age group, usually restricting exclusively either to the household or the school environment, but not both concomitantly as it was performed in this study.

The results from the statistical analysis support that WASH services and infrastructure, both in schools and homes, are essential to schoolchildren health, with regard to STHs. Filtered water in schools was indicated as a protective factor against parasitic infections caused by *A.lumbricoides* and *T.Trichiura.* While the presence of adequate sanitation in schools was associated with ascariasis prevalence, adequate sanitation at the household level was associated with hookworm infections, both promoting beneficial effects. However, sanitary interventions will not be fully effective in the prevention of STH infections without the promotion of access to quality public services for the population, particularly to the people living in poverty, the most vulnerable group.

Our findings reinforce the need of integrating the access to safe water, sanitation and adequate hygiene (WASH) services as part of the STHs control strategy. Special attention has to be devoted to the improvement of WASH services in schools, not just in households.

Although the collection of sample data covering the entire Brazilian territory is very complex, new research similar to the National Survey should be conducted, for monitoring the effects over time, with regard to the improvement of WASH services and infrastructure (house and schools), on the prevalence of infectious diseases.

## Supplementary Information

Additional file 1. Raw Database. Database containing all variables (outcome and explanatory) used in the study.

Additional file 2. Description of the National Survey on the Prevalence of Schistosomiasis mansoni and Soil-transmitted helminth infections (2011-2015). Detailed description of the National Survey.

## Data Availability

The dataset supporting the conclusions of this article is included within the article (and its additional files).

## Abbreviations

STH: Soil-transmitted helminth
WASH: Water, Sanitation and Hygiene
RR: Rate Ratio
BIC: Bayesian Information Criteria
AIC: Akaike Information Criteria
BFP: *Bolsa Família* Program
HRtWS: Human Right to Water and Sanitary Sewage
