# Supplementary material for: Association between water and sanitation and soil-transmitted helminthiases: Analysis of the Brazilian National Survey of Prevalence (2011–2015)
Source: Arch Public Health. 2021 May 19;79:83. doi: 10.1186/s13690-021-00602-7 (PMC8132366; doi:10.1186/s13690-021-00602-7)
Supplement: Supplementary file 2 — Description of the National Survey on the Prevalence of Schistosomiasis mansoni and Soil-transmitted helminth infections (2011-2015). Detailed description of the National Survey. [file 13690_2021_602_MOESM2_ESM.docx]

**Supplementary Material – Description of the National Survey on the Prevalence of Schistosomiasis mansoni and Soil-transmitted helminth infections (2011-2015)**

The National Survey on the Prevalence of Schistosomiasis mansoni and Soil-transmitted helminth infections (2011 – 2015) in Brazil was the first prevalence survey conducted at the nationwide scale, i.e. covering all the 27 Brazilian Federation Units (1). It was a population-based cross-sectional study with the objective of identifying the current prevalence of schistosomiasis mansoni, trichuriasis, hookworm and ascariasis, in students from 7 to 17 years old. The intention was also to verify if the prevalence of schistosomiasis mansoni in endemic areas were lower than that observed in the surveys conducted by Pellon and Teixeira (2) and by the Special Control Program for Schistosomiasis - PECE (3). For this purpose, 197,564 students living in 521 municipalities distributed in all five geographical regions of the country were examined. The age range (7-17 years old) was strategically chosen to cover schoolchildren who were at higher risk of contracting infections due to STH and schistosomiasis mansoni.

Students were invited to provide a stool sample for examination and the consent for participation was obtained from the parents of the schoolchildren. All children with positive result were sent to a health unit care to be treated. The survey data is public available and it can be fully accessed at the SUS Department of Informatics (DATASUS) web page ([www.datasus.gov.br](http://www.datasus.gov.br)).

Data from the National Survey were collected according to the follow sampling strategy:

(i) Brazilian municipalities were stratified into three categories according to their endemic level of schistosomiasis mansoni (non-endemic, low and high endemicity) and four population sizes (below 20,000 inhabitants, 20,000 - 150,000 inhabitants, 150,000 - 500,000 inhabitants, above 500,000 inhabitants);

(ii) in each stratum municipalities, schools (both private and public) per municipality and school classes per institution were randomly drawn to participate;

(iii) all students present in the classrooms on the day of the visit of the survey agents were invited to provide material for the parasitological examination of feces which was performed by using the Kato-Katz diagnostic method (4).

Only schoolchildren who delivered the fecal material required for parasitological examination, in good conservation conditions, were considered as sample elements.

Although the STH prevalence estimation were part of the study, the sample size calculations were performed using previous prevalence values of schistosomiasis mansoni as a reference. For the endemic area in the municipalities with less than 500 thousand inhabitants the sampling error of 5% and power of 90% were used to determinate the sample size. For non endemic areas and endemic areas with 500 thousand inhabitants or more, the sampling error was of 5% and power of 80%.

The sample size initially foreseen in the National Survey was 220,000 schoolchildren aged from 7 to 17 years, of both sexes, sampled in 541 municipalities distributed in the 27 Federation units of Brazil. However, the final sample was composed by in 521 municipalities (96.1% of planned) and 197,564 students (89.8% of planned).

The National Survey was approved by the Ethics and Research Committee of the René Rachou Research Center – Oswaldo Cruz Foundation and the National Research Ethics Commission – CONEP.

All the information provided in this supplementary material was retrieved from Katz (1).

**References**

1. Katz N. Inquérito Nacional de Prevalência da Esquitossomose Mansoni e Geo-helmintoses. Belo Horizonte, CPqRR; 2018. [https://www.arca.fiocruz.br/handle/icict/25662.](https://www.arca.fiocruz.br/handle/icict/25662.%20Accessed%2023%20Aug%202018) .
2. Pellon AB, Teixeira I. Distribuição da esquistossomose mansônica no Brasil. Divisão de Organização do Ministério da Saúde. Rio de Janeiro.1950.
3. Ministério da Saúde. SUCAM. Levantamento Nacional de Prevalência da esquistossomose mansoni, 1975-1979. Programa de Controle da Esquistossomose. Brasília. 1976. 41 p.
4. Katz N, Chaves A, Pellegrino J. A simple device for quantitative stool thick-smear technique in schistosomiasis mansoni. Revista do Instituto de Medicina Tropical de São Paulo. 1972; 4:397-400
